# Models of Acetylcholine and Dopamine Signals Differentially Improve Neural Representations

**DOI:** 10.3389/fncom.2017.00054

**Published:** 2017-06-22

**Authors:** Raphaël Holca-Lamarre, Jörg Lücke, Klaus Obermayer

**Affiliations:** ^1^Neural Information Processing Group, Fakultät IV, Technische Universität BerlinBerlin, Germany; ^2^Bernstein Center for Computational NeuroscienceBerlin, Germany; ^3^Cluster of Excellence Hearing4all and Research Center Neurosensory Science, Carl von Ossietzky Universität OldenburgOldenburg, Germany; ^4^Machine Learning Lab, Department of Medical Physics and Acoustics, Carl von Ossietzky Universität OldenburgOldenburg, Germany

**Keywords:** acetylcholine, dopamine, neuromodulator, sensory representations, neural networks, biology-inspired learning, representation learning

## Abstract

Biological and artificial neural networks (ANNs) represent input signals as patterns of neural activity. In biology, neuromodulators can trigger important reorganizations of these neural representations. For instance, pairing a stimulus with the release of either acetylcholine (ACh) or dopamine (DA) evokes long lasting increases in the responses of neurons to the paired stimulus. The functional roles of ACh and DA in rearranging representations remain largely unknown. Here, we address this question using a Hebbian-learning neural network model. Our aim is both to gain a functional understanding of ACh and DA transmission in shaping biological representations and to explore neuromodulator-inspired learning rules for ANNs. We model the effects of ACh and DA on synaptic plasticity and confirm that stimuli coinciding with greater neuromodulator activation are over represented in the network. We then simulate the physiological release schedules of ACh and DA. We measure the impact of neuromodulator release on the network's representation and on its performance on a classification task. We find that ACh and DA trigger distinct changes in neural representations that both improve performance. The putative ACh signal redistributes neural preferences so that more neurons encode stimulus classes that are challenging for the network. The putative DA signal adapts synaptic weights so that they better match the classes of the task at hand. Our model thus offers a functional explanation for the effects of ACh and DA on cortical representations. Additionally, our learning algorithm yields performances comparable to those of state-of-the-art optimisation methods in multi-layer perceptrons while requiring weaker supervision signals and interacting with synaptically-local weight updates.

## 1. Introduction

Neurons in the cortex represent countless features of sensory signals, from the frequencies of photons falling on the retina to high-level attributes like quantities and numbers. The particular form a sensory representation takes is critical to perception. For instance, experienced musicians display enhanced sensory representations which putatively explain their finer perceptual abilities (Elbert et al., 1995; Pantev et al., 1998, 2001). This view is further supported by the observation that, following discrimination training, improvements in perceptual sensitivity correlate with the degree of reorganization in cortical representations (Recanzone et al., 1992, 1993; Weinberger, 2003; Polley et al., 2006). On the other hand, perceptual disorders like phantom limb pain (Ramachandran et al., 1992; Halligan et al., 1993; Flor et al., 2006) or tinnitus (Eggermont and Roberts, 2004) appear to be correlates of degenerate sensory representations.

In animals, sensory representations undergo modifications in various circumstances, for instance following extensive perceptual training (Weinberger and Bakin, 1998; Harris et al., 2001; Schoups et al., 2001; Fletcher and Wilson, 2002; Fritz et al., 2003; Wang et al., 2003; Bao et al., 2004; Yang and Maunsell, 2004; Polley et al., 2006; Poort et al., 2015), repeated sensory exposure (Han et al., 2007; Kim and Bao, 2009), cortical stimulation, (Godde et al., 2002; Dinse et al., 2003; Tegenthoff et al., 2005), or sensory deprivation (Calford and Tweedale, 1988; Allard et al., 1991; Gambino and Holtmaat, 2012). Additionally, the neuromodulators acetylcholine (ACh) and dopamine (DA) bear potent effects on cortical representations. In particular, repeated efflux of either ACh (Kilgard and Merzenich, 1998a; Froemke et al., 2007, 2013; Gu, 2003; Weinberger, 2003) or DA (Bao et al., 2001; Frankó et al., 2010) coinciding with a stimulus strengthens the responses of neurons to this stimulus and enlarges its cortical representation.

ACh and DA are critical to forms of learning which require modifications of sensory representations. For instance, lesion of the cholinergic (Butt and Hodge, 1995; Fletcher and Wilson, 2002; Conner et al., 2003; Wilson et al., 2004; Conner et al., 2010) or dopaminergic (Kudoh and Shibuki, 2006; Molina-Luna et al., 2009; Hosp et al., 2011; Luft and Schwarz, 2009; Schicknick et al., 2012) system disrupts perceptual and motor learning as well as the associated plasticity in cortical maps. These observations suggest that the neuromodulators orchestrate plastic changes that refine cortical representations and give rise to perceptual and motor learning.

In physiological conditions, ACh transmission appears to signal attentional effort, a construct reflecting both the relevance and difficulty of a task (Himmelheber et al., 2000; Arnold et al., 2002; Kozak et al., 2006; Sarter et al., 2006). DA carries information relative to reward-prediction errors (RPEs) (Schultz et al., 1997; Schultz, 2007, 2010). Although their release properties are relatively well defined, the functional roles these signals serve in shaping neural representations is unclear.

Much like the cortex, artificial neural networks (ANNs) represent input data in the form of neural activation. As for other machine learning algorithms, the performance of ANNs critically depends on the representation data take. The most widely used learning rule for ANNs, the error back-propagation algorithm (Werbos, 1974; Rumelhart et al., 1985), learns representations optimised for specific tasks. Although, the back-propagation algorithm yields remarkable performances, it is unlikely to be implemented in biological neural structures and it also bears its own limitations. For instance, in order to compute the error function, a target output must be specified for each training example, making training data expensive to acquire. Additionally, weight updates require information not available locally at the weights which limits the use of the back-propagation algorithm in physical devices like neuromorphic chips.

In the present work we explore the use of signals inspired from ACh and DA for learning in a neural network model. This effort serves two aims: first, to shed light on the functional roles of ACh and DA in shaping cortical representations and, second, to provide inspiration for novel training methods for ANNs.

Previous studies examine the roles of ACh and DA in neural information processing. Weinberger and Jonathan (1998) develop a model of ACh signaling to investigate its function in classical conditioning. Li and Cleland (2013) present a detailed biophysical model of ACh neuromodulation in the olfactory bulb. However, these studies do not see to the perceptual benefits of long-term plasticity induced by ACh. Other work tackle the question of DA-modulated plasticity in neural networks. Roelfsema and colleagues show that a signal inspired from DAergic signaling allows a network to learn various classification tasks (Roelfsema and Ooyen, 2005; Roelfsema et al., 2010; Rombouts et al., 2012). Similarly, other models make use of DA-like reinforcement signals to learn stimulus-response associations (e.g., Law and Gold, 2009; Liu et al., 2010). In these cases, however, the models for the plastic effects of DA were chosen to carry out reinforcement learning rather than to tally with experimental observations.

In contrast with previous work, we base our modeling effort on the well-documented observation that pairing ACh or DA release with a stimulus boosts neural responses to the stimulus. We use this model to study the perceptual benefits of ACh- and DA-induced plasticity under natural release conditions. In more details, we make use of a Hebbian-learning neural network and simulate the physiological release schedules of ACh and DA. In the model, ACh activation approximates attentional demand while DA activation arises from RPEs. We find that the neuromodulators trigger distinct changes in representations that both improve the network's classification performance. Specifically, ACh leads to changes in synaptic weights such that more neurons are dedicated to stimuli that are challenging for the network. DA adapts synaptic weights to the reward contingencies of a task, thereby sharpening neural tuning with respect to the classes of the task. These results provide a functional explanation for the roles of cholinergic and dopaminergic signals in refining cortical representations.

Our learning algorithm offers several advantages from a practical perspective. First, the network achieves performances comparable to those of state-of-the-art optimisation methods used to train multi-layer perceptrons (MLPs) while requiring weaker supervision signals. Second, learning takes place even in the absence of environmental feedback. And third, weight updates are based on synaptically-local information and on two signals broadcasted identically to all neurons. These features may make the algorithm interesting for functional applications such as learning in neuromorphic processors.

## 2. Methods

### 2.1. Hebbian network model

For our study, we make use of a Hebbian-learning neural network model introduced by Keck et al. (2012). The learning mechanisms implemented in this model achieve approximately optimal learning in terms of maximum likelihood estimation (see original publication for a detailed discussion). As a theoretically well-founded and biologically realistic model, this network is a natural starting point for our work. In this section, we briefly present the original model and then describe our simulation of the neuromodulators ACh and DA.

**Figure 1:**
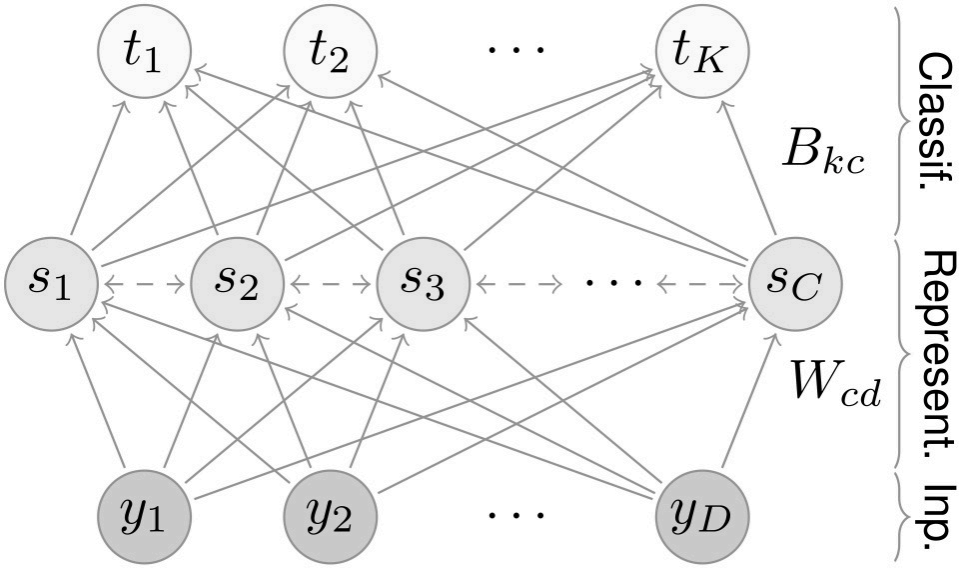
Network architecture. The network contains three layers: an input, a representation, and a classification layer. For the MNIST dataset, the input and classification layers contain *D* = 28 × 28 = 784 and *K* = 10 neurons, respectively. The number of representation neurons is variable; for most results we use *C* = 7 × 7 = 49 neurons.

The network consists of three layers, an input, a representation, and a classification layer (Figure 1). Input values activate neurons in the first layer; activity then propagates through the network in the following steps.

#### 2.1.1. Feedforward inhibition

In mammals, the responses of sensory neurons are largely invariant to contrast in sensory stimuli (Sclar et al., 1990; Stopfer et al., 2003; Mante et al., 2005; Assisi et al., 2007; Olsen and Wilson, 2008), in part due to rapid feedforward inhibition (Pouille and Scanziani, 2001; Swadlow, 2003; Mittmann et al., 2005; Wehr and Zador, 2005; Pouille et al., 2009; Isaacson and Scanziani, 2011). To emulate this process, neural activations in the input layer are normalized:

(1) $$\begin{array}{r} y_{d}=\left(A-D\right)\frac{{ỹ}_{d}}{\sum_{d\prime =1}^{D}{ỹ}_{d\prime }}+1, \end{array}$$

where $\vec{ỹ}$ are input data, *A* is a normalization constant, and *D* is the number of input neurons. This form of normalization yields contrast-invariant responses in representation neurons. For the dataset used in this work, *D* = 28 × 28 = 784 input neurons. For other hyper-parameters, values are determined through grid search to maximize classification performance (see Table A1 in Appendix section).

#### 2.1.2. Input integration

Neurons in the representation layer integrate their input through a weighted sum:

(2) $$\begin{array}{r} I_{c}=\overset{D}{\underset{d=1}{\sum }}S\left(W_{cd}\right)y_{d}, \end{array}$$

where *W* is the weight matrix between the input and representation layers and *S*(·) is a linearised logarithm function given by:

(3) $$\begin{array}{l} S\left(W_{cd}\right)=\left\{ \begin{array}{cc} W_{cd}\; & \text{if }W_{cd}<1\\ \log\left(W_{cd}\right)+1\; & \text{if }W_{cd}\geq 1. \end{array}\right. \end{array}$$

Taking the logarithm of *W*_*cd*_ guarantees approximate optimal learning of the weights, with the linearisation ensuring that the function is never negative for *W*_*cd*_ ≥ 0.

#### 2.1.3. Lateral inhibition

The integrated input is fed through a softmax function that models global lateral inhibition:

(4) $$\begin{array}{r} s_{c}=\frac{\exp\left(I_{c}\right)}{\sum_{c\prime }\exp\left(I_{c\prime }\right)}. \end{array}$$

#### 2.1.4. Hebbian learning

Hebbian learning takes place between the input and representation neurons:

(5) $$\begin{array}{r} \Delta W_{cd}=\epsilon \cdot \left(s_{c}y_{d}-s_{c}W_{cd}\right), \end{array}$$

where ϵ is the learning rate.

#### 2.1.5. Classification

We subject the network to a classification task of images of hand-written digits from the MNIST dataset (LeCun et al., 1998b). These input images provide stimuli of intermediate complexity and high-dimensionality akin to natural sensory stimuli, making them a popular dataset to study neural information processing (Nessler et al., 2013; Schmuker et al., 2014). These data consist of gray-scale images with pixel values in the range [0, 255] fed as input $\vec{ỹ}$ to the first layer.

In the classification layer, we use statistical inference to decode activity in the representation layer. Given an input pattern $\vec{y}$ and the model parameters Θ, we want to infer the class of the input pattern, that is, to compute the posterior $\mathrm{Pr}\left(k|\vec{y},\Theta \right)$. Here, we approximate the posteriors using the labels of the input images. We first compute a value *B*_*kc*_:

(6) $$\begin{array}{r} B_{kc}\;:=\frac{1}{N_{m}}\overset{N_{m}}{\underset{n=1}{\sum }}\mathrm{Pr}\left(c|{\vec{y}}^{\left(n\right)},W\right)=\frac{1}{N_{m}}\overset{N_{m}}{\underset{n=1}{\sum }}s_{c}^{\left(n\right)}, \end{array}$$

with *N*_*m*_ input patterns ${\vec{y}}^{\left(n\right)}$ bearing a label *m* = *k*. The matrix *B* can be interpreted as the weights between the representation and classification layers. This matrix is updated after every presentation of 100 images, or roughly 600 times during one iteration over the dataset. The posteriors are approximated as:

(7) $$\begin{array}{r} \mathrm{Pr}\left(k|\vec{y},\Theta \right)\approx t_{k}=\overset{C}{\underset{c=1}{\sum }}\frac{B_{kc}s_{c}}{\sum_{k\prime =1}^{K}B_{k\prime c}}. \end{array}$$

As a classification result $\hat{m}$, we take the unit with the largest value of approximation to the posterior:

(8) $$\begin{array}{r} \hat{m}=\overset{K}{\underset{k=1}{\text{argmax}}}\left(t_{k}\right). \end{array}$$

This hierarchical formulation allows to decode activity in the representation layer, providing a probabilistic classification of the input images.

Previous work based on a fully probabilistic description of the Hebbian-learning network model (Forster et al., 2016; Forster and Lücke, 2017) shows that local Hebbian learning converges to the weight matrix *B* without requiring the non-local summation over *k*. This is true also when using a small fraction (≈1%) of labeled training examples. Learning the classification weights can therefore be achieved while respecting biological constraints. For this work, we mainly focus on the standard fully labeled setting, as is customary (Keck et al., 2012; Nessler et al., 2013; Schmuker et al., 2014; Diehl and Cook, 2015; Neftci et al., 2015), but also provide results for experiments with very few labels.

### 2.2. Model of the neuromodulators

#### 2.2.1. Effects on plasticity

We extend the network model described above to emulate the effects of ACh and DA on neural representations. Specifically, we simulate the impact of the neuromodulators as a modulation of the network's learning rate:

(5a) $$\text{acetylcholine}:\;\Delta W_{cd}\;=\;\epsilon \;\cdot \;ACh\;\cdot \;(s_{c}y_{d}-s_{c}W_{cd}),$$

(5b) $$\text{dopamine}:\;\Delta W_{cd}\;=\;\epsilon \cdot DA\cdot (s_{c}y_{d}-s_{c}W_{cd}),$$

where *ACh* and *DA* represent the activation of the corresponding neuromodulatory system. This model is in general agreement with experimental observations in that both ACh (Bröcher et al., 1992; Chun et al., 2013) and DA (Blond et al., 2002; Sun et al., 2005; Matsuda et al., 2006) are reported to promote synaptic plasticity. This model for the neuromodulators was chosen so as to reproduce the results of pairing experiments in mammals (see Results section).

#### 2.2.2. Acetylcholine and attentional efforts

ACh release in the mammalian neocortex is tightly linked with attentional processes. For instance, as rats detect a behaviorally meaningful sensory cue, a spike in cortical ACh accompanies the reorientation of their attention towards the cue (Parikh et al., 2007). Additionally, when rats perform a task requiring sustained attention, the concentration of ACh in their prefrontal cortices more than doubles compared to control (Arnold et al., 2002; Kozak et al., 2006). In the course of such tasks, distractors that further tax the animals' attentional systems trigger supplemental ACh release (Himmelheber et al., 2000; Kozak et al., 2006). These observations indicate that the cholinergic system responds to events demanding an animal's attention such as relevant stimuli or challenging tasks. In this sense, ACh transmission reflects the cognitive construct of attentional effort defined as a subject's motivated effort to maintain performance under challenging conditions (Sarter et al., 2006).

**Figure 2:**
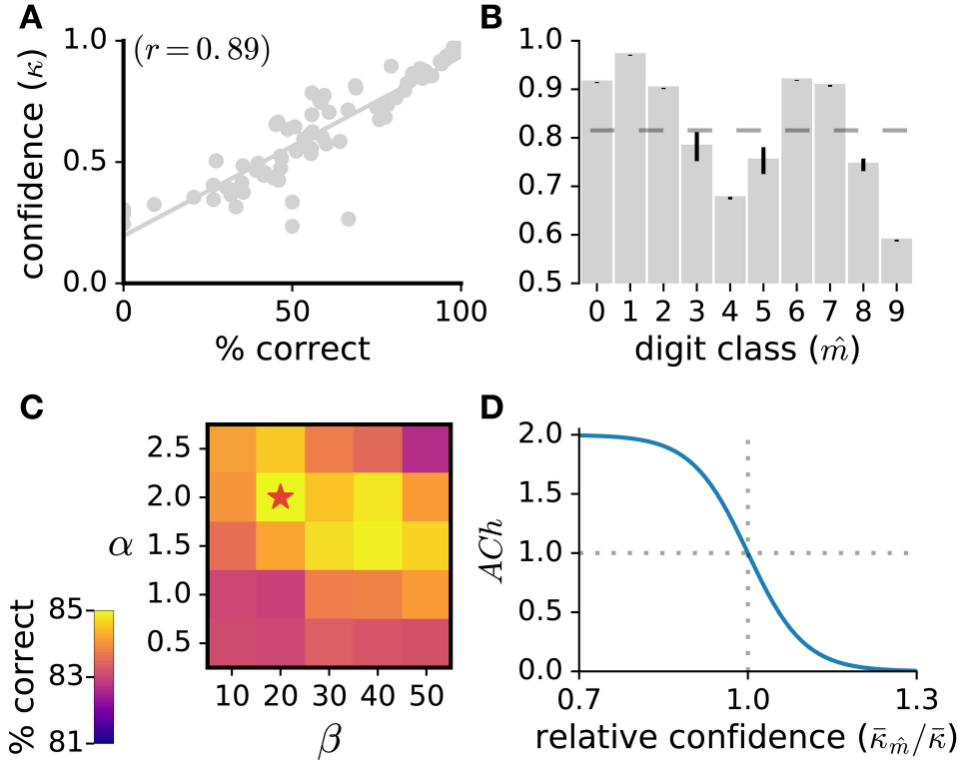
**(A)** Classification confidence strongly correlates with classification accuracy. Here, we measure the network's classification confidence for the test images of the MNIST dataset, bin the classification confidence (bin size of 0.02%) and calculate the average correct classification for each bin. **(B)** Average classification confidence ${\overset{-}{\kappa }}_{\hat{m}}$ for the 10 digit classes, with the mean confidence over all classes $\overset{-}{\kappa }$ indicated as a dashed line. Data are the mean of 10 runs, error bars indicate the standard deviation across runs. **(C)** Parameter exploration for the α and β parameters of the ACh release function. A star indicates the parameter set yielding maximal accuracy. **(D)** ACh activation function (Equation 10) taking as input the relative confidence ${\overset{-}{\kappa }}_{\hat{m}}/\overset{-}{\kappa }$. This ratio quantifies the demand of the current stimulus.

In the present work, we model ACh activation to approximate attentional demand. To quantify how demanding a stimulus is for the network, we use the network's classification confidence. Classification confidence is measured as the classifier's maximal posterior over the digit classes, $\kappa =\overset{K}{\underset{k=1}{\max}}\left(t_{k}\right)$. Classification confidence strongly correlates with classification accuracy (*r* = 0.89, Figure 2A) indicating that this measure is suitable to quantify stimulus demand. For each stimulus, the value of the *ACh* variable is given by:

(10) $$\begin{array}{r} ACh=\frac{\alpha }{1.0+\exp\left(\beta \cdot \left({\overset{-}{\kappa }}_{\hat{m}}/\overset{-}{\kappa }-1.0\right)\right)} \end{array}$$

where ${\overset{-}{\kappa }}_{\hat{m}}$ is the network's average classification confidence for the inferred class of the current stimulus, $\overset{-}{\kappa }$ is the average classification confidence for all stimuli, and α and β are hyper-parameters of the sigmoid function whose values are determined through grid search (Figure 2). According to this formulation, the lower the classification confidence (i.e., the greater the stimulus difficulty), the larger the ACh activation. Note that, to compute the average classification confidence over the digit classes, we use the network's inferred classification ($\hat{m}$) and not the stimulus label. Thus, for a given stimulus, ACh activation is evaluated without requiring immediate environmental information. Also note that the classification confidence for the same stimulus may vary during training as the network's weight matrices *W* and *B* are updated.

#### 2.2.3. Dopamine and reward prediction errors

**Figure 3:**
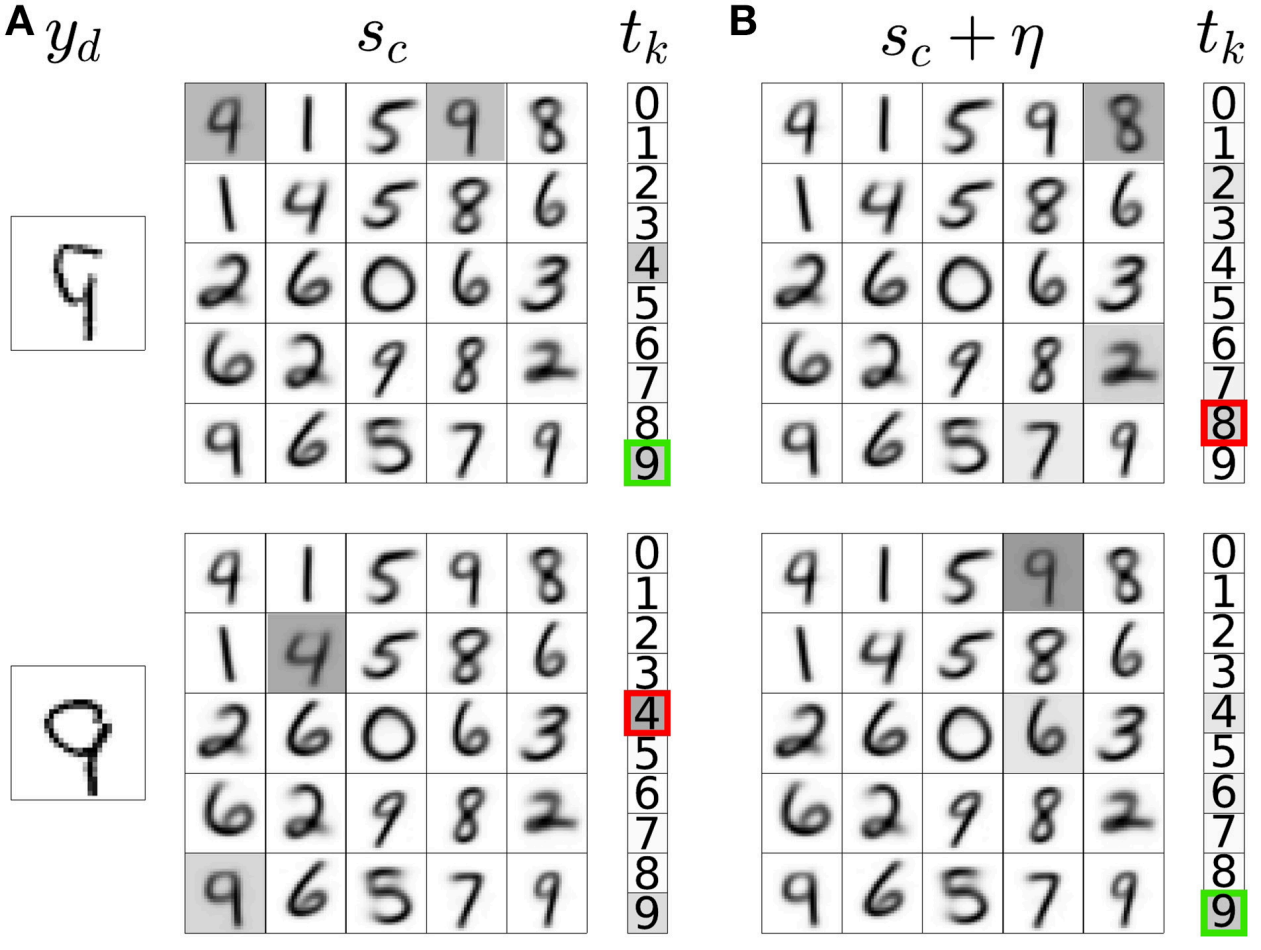
Noisy neural activation in the representation layer allows explorative classification decisions. **(A)** Activations of input neurons (*y*_*d*_), weights of a subset of representation neurons with their corresponding activations prior to noise injection (*s*_*c*_, gray highlights), and activations of classification neurons (*t*_*k*_) with the network's classification output indicated as a bold colored outline. The example input images are correctly (top) and incorrectly classified (bottom). **(B)** Noise addition in the activations of representation neurons leads to incorrect (top) and correct (bottom) explorative classification decisions. In these two different outcomes of exploration, the variable *DA* in Equation 5b takes a distinct value (δ_−/−_ and δ_−/+_, respectively).

DA efflux in animals follows RPEs (Schultz et al., 1997; Satoh et al., 2003; Tobler et al., 2005; Schultz, 2010). We reproduce this release schedule in the model as follows. First, we allow explorative decision making by injecting additive noise in the activation of representation neurons (Figure 3):

$$\begin{array}{ll} I_{c}=\overset{D}{\underset{d=1}{\sum }}S\left(W_{cd}\right)y_{d}+\eta_{c},\; & \;\\ \eta_{c}\sim N\left(0,\upsilon \right), \end{array}$$

where N is a normal distribution with zero mean and variance υ. This method for exploration approximates the softmax rule for action selection in reinforcement learning (Sutton and Barto, 1998). Following this rule, actions are selected stochastically with the probability of selecting an action proportional to its expected reward. The parameter υ corresponds to the temperature parameter of the softmax rule: for υ → ∞, all classification decisions have equal probabilities; for υ → 0^+^, classification is purely exploitative. We find the optimal value for υ through grid search.

We then compute the classification output for each $\vec{y}$ with and without the addition of noise η. If noise addition results in a classification decision that is different from the decision without noise addition, the classification is labeled as explorative; otherwise it is labeled as exploitative. If the network takes an exploitative decision it is said to predict a reward (+*pred*); if it takes an explorative decision it is said to not predict a reward (−*pred*). The network is rewarded for taking correct classification decisions (+*rew*) and not rewarded for incorrect decisions (−*rew*). The difference between the predicted and delivered rewards gives rise to a RPE. There are four possible RPE scenarios. In each of these cases, the *DA* variable in Equation 5b takes a distinct value:

(12) $$\begin{array}{r} DA=\left\{ \begin{array}{cc} \delta_{+/+}\; & \;\text{if}+\text{pred }\text{and}+\text{rew}\\ \delta_{+/-}\; & \;\text{if}+\text{pred }\text{and}-\text{rew}\\ \delta_{-/+}\; & \;\text{if}-\text{pred }\text{and}+\text{rew}\\ \delta_{-/-}\; & \;\text{if}-\text{pred }\text{and}-\text{rew}\\ \; \end{array}\right. \end{array}$$

where δ_./._ are constants whose values are determined through 4-dimensional parameter search to maximize classification performance.

#### 2.2.4. Critical period

We are interested in changes in sensory representations triggered by neuromodulators in adult animals. Adult animals possess stable neural representations of their environment learned in early life during a brief window of heightened plasticity. During this so-called critical period, the response properties of neurons rapidly adjust to the statistical structure of sensory stimuli (Sengpiel et al., 1999; de Villers-Sidani et al., 2007; Han et al., 2007; Barkat et al., 2011).

As a model of this critical period, we pre-train the network solely through Hebbian learning (Equation 5). The network then learns synaptic weights based on correlations in the activation of input neurons, with weights that resemble the different digit classes. The weights in the representation layer are then learned solely through the statistics of the input images and do not reflect the task to be performed. As learning progresses, performance on the classification task increases and eventually saturates. Once performance reaches a plateau, we allow the release of ACh or DA. As an additional control condition, we also continue training the network through Hebbian learning. Omitting the pre-training results in the same functional performance but, without it, the optimal DA activation values found through parameter search differ (see **Figure 6**).

## 3. Results

### 3.1. Pairing experiment

**Figure 4:**
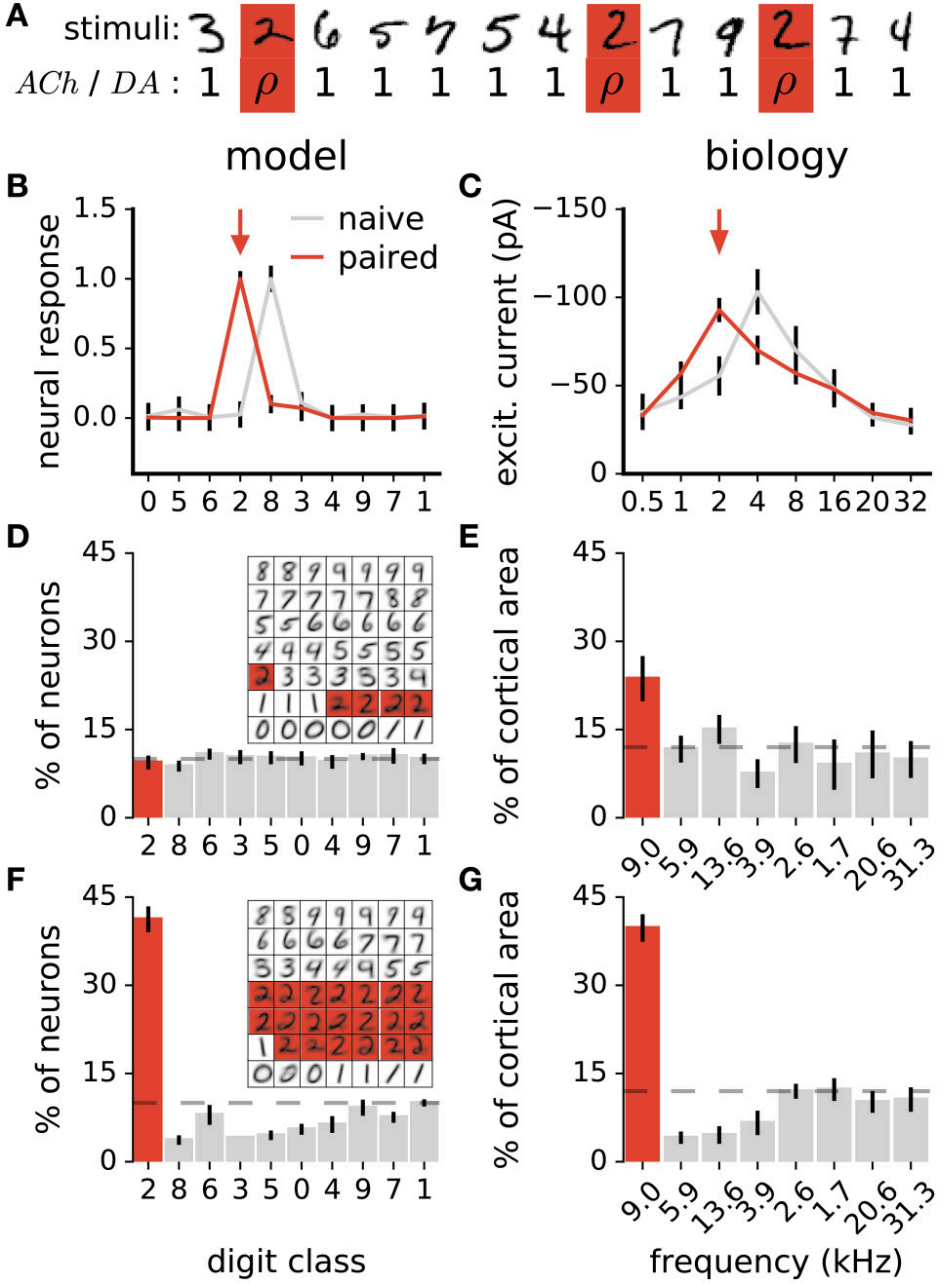
Stimulus pairing with ACh or DA enhances the stimulus' representation. **(A)** Simulation of the neuromodulator-stimulus pairing protocol. The *ACh* or *DA* variables in Equations 5a or 5b is set to a constant value ρ > 1 for stimuli of the paired class (“2”, in this case) and to 1 for all other classes. **(B)** Mean responses of a neuron to the digit classes. Traces are before and after pairing images labeled as “2” (arrow) with neuromodulator activation ρ = 20. Classes are ordered with their distance from class “0” (see text for details). Error bars are a standard deviation. **(C)** Synaptic tuning curves of a neuron in the rat primary auditory cortex. Traces are before and after a 2 kHz tone (arrow) is paired with high-frequency electrical stimulation of the nucleus basalis triggering ACh release. Error bars are the standard error of the mean. Reproduced from Froemke et al. (2007), with permission. In the model and in animals, the pairing procedure boosts responses of individual neurons to the paired stimulus. **(D,F)** Histogram of class preferences in the network model before **(D)** and after **(F)** the pairing manipulation. Classes are ordered with their distance from the paired class. Dashed line is a uniform distribution, data are the mean of 10 runs, error bars indicate a standard deviation. Inset: weights of the representation neurons for an example network; highlights indicate neurons whose preferred classes are “2”. **(E,G)** Histogram of best frequencies in the auditory cortices of rats before **(E)** and after **(G)** a 9 kHz tone is paired with stimulation of midbrain dopaminergic neurons. Frequencies are ordered to their difference from the paired tone. Modified from Bao et al. (2001), with permission. In the simulation as in biology, the pairing protocol enhances the representation of the paired stimulus and suppresses that of neighboring ones.

In animals, coupling a stimulus with the release of either ACh (Kilgard and Merzenich, 1998a; Weinberger, 2003; Froemke et al., 2007, 2013) or DA (Bao et al., 2001; Frankó et al., 2010) triggers long-lasting changes in sensory representations. Specifically, sensory neurons increase their responses to the paired stimulus, resulting in more neurons preferring this stimulus. To test whether our model of ACh and DA is in agreement with this observation, we perform a similar experiment. The experiment consists of coupling all stimuli of a target class with *ACh* or *DA* = ρ in Equations 5a or 5b, where ρ is a constant >1 (Figure 4A). Stimuli of all other classes have *ACh* and *DA* = 1. We then examine the distribution of class preferences in the network. The preferred digit class of a neuron is determined by taking ${\text{argmax}}_{k=1}^{K}\left(B_{kc}\right)$ which gives the class to which neuron *c* maximally responds to. We find that the pairing protocol increases the responses of individual neurons to the paired stimulus class and augments the number of neurons preferring this class, in agreement with experimental data (Figures 4B–G). Furthermore, the procedure reduces the number of units tuned to classes close to the paired one (class closeness is measured as the Euclidean distance between the averages of all training examples of each class). These findings are in line with pairing experiments with DA showing that the cortical representations of frequencies neighboring a paired tone shrink as a result of the pairing procedure (Figure 4G; Bao et al., 2001). This observation however contrasts with pairing experiments with ACh which result in enlargements of the cortical representations of both the paired frequency and adjacent ones (Kilgard and Merzenich, 1998a). For this work, this difference in the effects of ACh and DA is not taken into account.

### 3.2. Physiological release schedule

#### 3.2.1. Optimal release values

**Figure 5:**
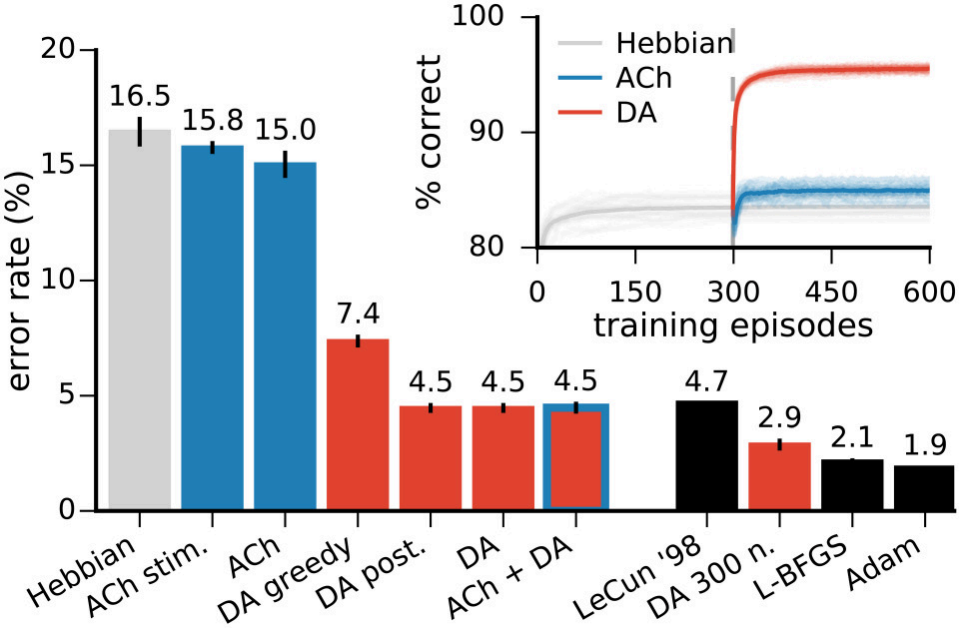
Neuromodulator release improves the network's classification performances. Left bar plots: error rates on the MNIST test dataset in networks with 49 representation neurons. All approaches with neuromodulators lead to significant improvements over Hebbian learning alone. “ACh stim.” is for stimulus-wise ACh activation, “DA greedy” is for a network without exploration, “DA post.” is for a network using the classifier's posteriors as an approximation to the expected value of the reward (see text for details). DA alone and DA with ACh yield the best performance. Right bar plots: comparison with other training methods for MLPs. All results are for networks of the same architecture, namely a single hidden layer with 300 units. LeCun ‘98 are the original results from LeCun et al. (1998a) on the MNIST dataset. L-BFGS and Adam are optimisation methods for MLPs (see Appendix for details). Inset: progression of the test performance for the networks with 49 neurons. Darker traces are averages over 20 runs, lighter traces are individual runs. Data for the bar plots are the mean of 20 runs, error bars indicate a standard deviation.

**Figure 6:**
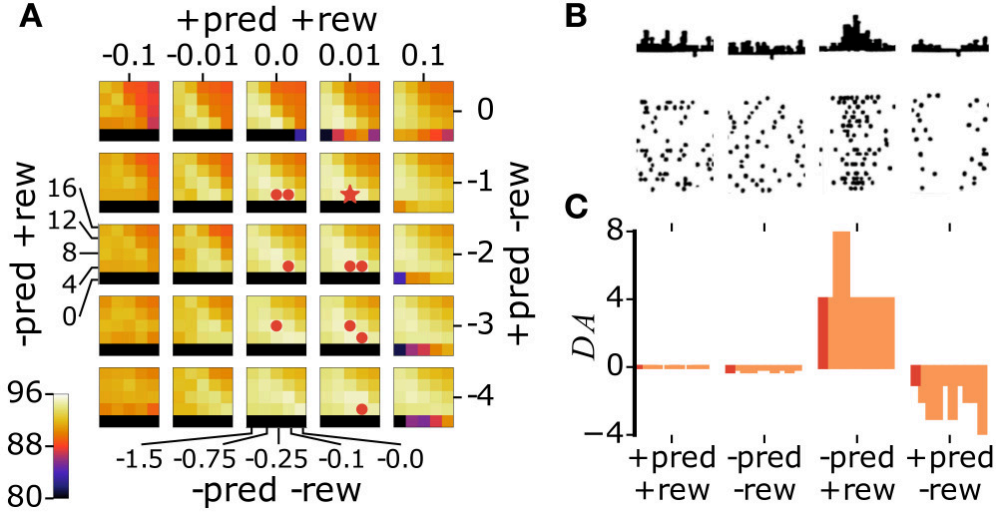
The model's optimal DA activation profile matches the one reported in mammals. **(A)** We explore different values for the four δ_./._ constants through grid search and report the classification performance of the network (colored axis, data are averages over 10 runs). A star indicates the best parameter set, dots indicate parameter sets yielding performances not statistically significantly different from that of the best set (*p* > 0.01). **(B)** Firing of dopaminergic neurons in monkeys in RPE scenarios equivalent to those of the model (modified from Schultz et al. (1997), with permission). **(C)** Bar plot of the best parameter set (dark red) and sets not significantly different from best (light red). The parameter sets are sorted in decreasing order of their classification accuracies, from left to right.

With our model in general agreement with the results of pairing experiments, we can now study the effects of the natural release schedules of ACh and DA. We first pre-train the network through Hebbian learning. As training progresses, performance saturates (Figure 5, inset). After this point, we allow the release of ACh or DA. We perform parameter search to identify the optimal values for parameters α and β in Equation 10 (Figure 2C) and for the δ_./._ constants in Equation 12 (Figure 6). In the case of the δ_./._ constants, we find that for surprising rewards (−*pred*, +*rew*) the optimal δ_−/+_ is positive while in the absence of an expected reward (+*pred*, −*rew*) the optimal δ_+/−_ is negative. For correctly predicted rewards (either +*pred*, +*rew* or *-pred*, −*rew*) the optimal δ_+/+_ and δ_−/−_ are close to zero. This optimal activation profile matches that observed in primates (Schultz et al., 1997; Tobler et al., 2005), Figures 6B–C).

#### 3.2.2. Effects of ACh

**Figure 7:**
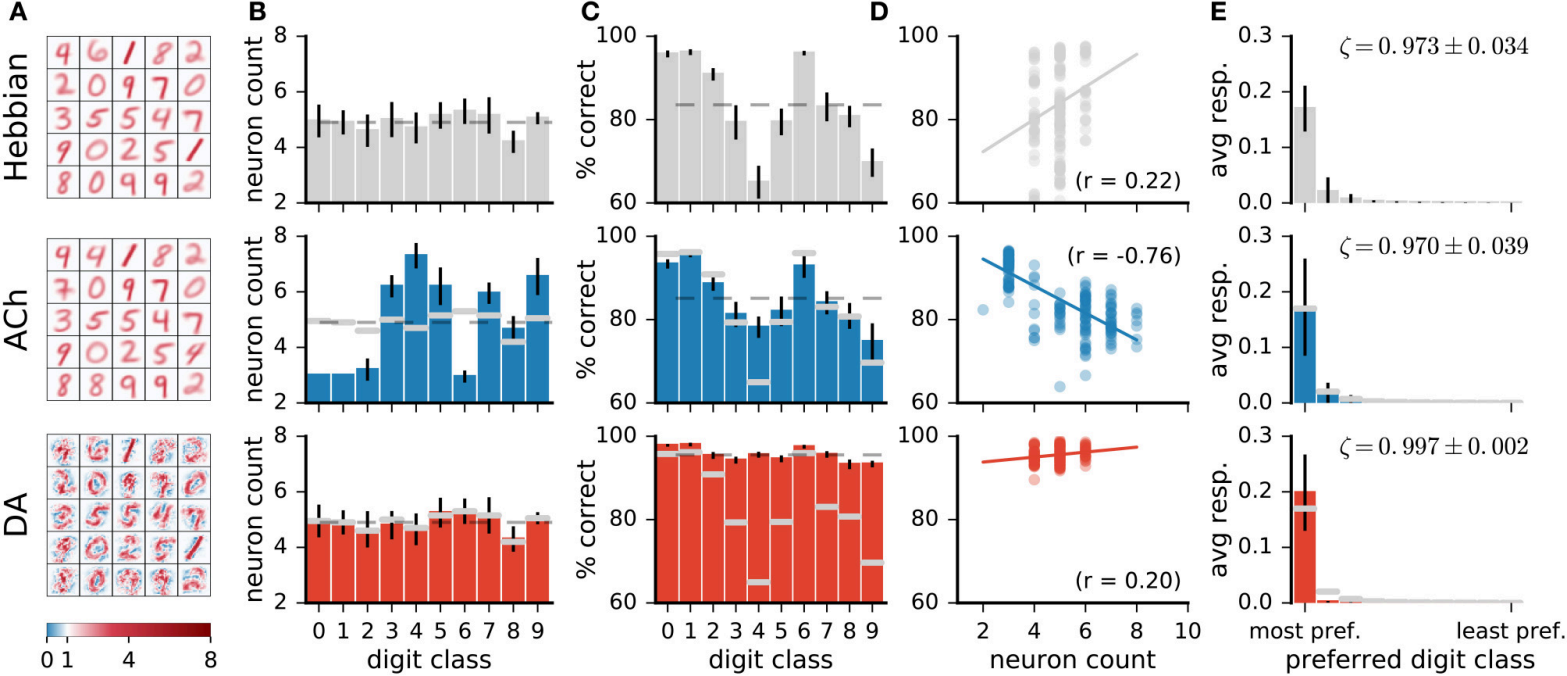
Network changes following neuromodulator release. **(A)** Weights of a subset of representation neurons (25 out of 49). **(B)** Histogram of class preferences. Dashed line is a uniform distribution. ACh increases the number of neurons preferring the more challenging classes. **(C)** Performance of the network on the different classes. Dashed line is average over all classes. **(D)** Performance on a class as a function of the number of neurons preferring this class. There is a positive correlation for Hebbian and DA-based learning. The learning mechanism in ACh reverses this correlation. **(E)** Average responses of neurons to the digit classes, with the classes ordered by the neurons' preference. ζ indicates mean neural selectivity (see text). DA sharpens the responses of neurons, enhancing their activations to their preferred classes and reducing their activations to non-preferred classes. Data are for 20 runs, error bars indicate a standard deviation. Gray overlaid bars in (**B,C**, and **E**) are values for Hebbian learning for comparison.

Visual inspection of the weights of the network (Figure 7A) indicates that ACh alters the number of neurons dedicated to the different digit classes. For instance, there are more neurons resembling a “4” and fewer neurons resembling a “1” after training with ACh. We quantify this redistribution by determining the preferred class of a representation neuron. For Hebbian learning, the distribution of preferred classes is close to uniform but not entirely so (Figure 7B). There is a positive correlation between the number of neurons dedicated to a class and the network's performance on this class (*r* = 0.22, Figure 7D), suggesting that representing a class with more neurons is beneficial to performance.

Training with ACh redistributes class preferences in the network, leading to a less uniform distribution. Specifically, ACh increases the number of neurons dedicated to challenging classes while easier classes are represented with fewer units. Consider for example the classes “1” and “4,” the stimuli on which the network performs best and worst, respectively (Figure 7C, top row). ACh release leads to a respective decrease and increase in the number of neurons preferring these classes (Figure 7B). The redistribution of neurons elicited by ACh raises the network's accuracy on the difficult classes (e.g., “4”) and lowers performance on the easy classes (e.g., “1,” Figure 7C, middle row). ACh thus reverses the correlation between neuron count and performance (*r* = −0.79, Figure 7D). On average over all classes, performance rises from 83.5 ± 0.7% with Hebb's rule alone to 85.0 ± 0.6% when supplemented with ACh, corresponding to a relative decrease of 12% in the error rate.

In addition to ACh activation computed as an average over the classes $\hat{m}$, we experiment with stimulus-wise ACh activation. Here, the value of the *ACh* variable is determined for each individual stimulus based on the classifier's posterior for this stimulus (specifically, we use the term κ instead ${\overset{-}{\kappa }}_{\hat{m}}$ in Equation 10). Although this approach also improves performance, the gains in accuracy are of smaller magnitude than if ACh activation is computed as an average over the classes (Figure 5, “ACh stim.”). We explain this outcome as the learning mechanism attributing a too great representational importance to demanding but detrimental data, for instance miss-labeled or outlier data points.

#### 3.2.3. Effects of DA

In contrast with ACh signalling, DA bears little effect on the number of neurons responsive to the different classes (Figure 7B). For both Hebbian and DA-based learning, the distribution of the neurons' preferred digit class is close to uniform. The positive correlation between neuron count and classification performance also remains after training with DA (Hebbian: *r* = 0.22, DA: *r* = 0.20).

**Figure 8:**
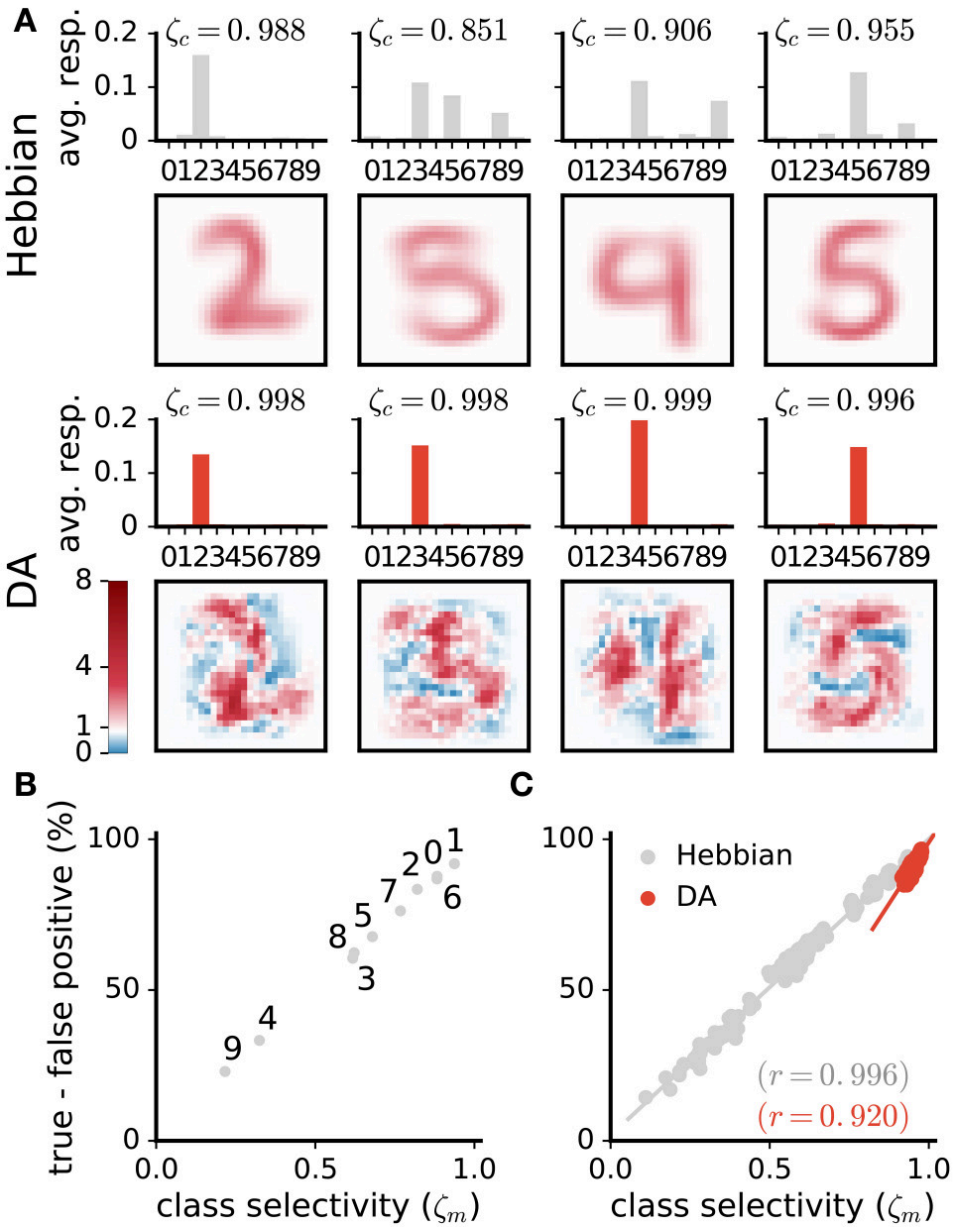
DA enhances class selectivity in neurons. **(A)** 1st and 3rd rows: average responses of example neurons to the different integer classes. 2nd and 4th rows: depictions of the neurons' weights. The color axis represents weight strength. While neurons in the Hebbian network respond to stimuli of multiple classes, those trained with DA respond almost exclusively to a single class. This observation is quantified as the selectivity ζ_*c*_ of neurons (Equation 13). **(B,C)** Difference between rates of true and false positives for each digit class as a function of a network's selectivity for this class. **(B)** Data are for a single Hebbian neural network. **(C)** Data are for 20 networks for both Hebbian- and DA-based learning. DA enhances neural selectivity which translates to greater classification accuracy.

Visual inspection of the weights suggests that DA makes neurons' weights more selective to specific digit classes. Consider the example weights shown in Figure 8A. Weights in one column are for corresponding neurons in a Hebbian and DA network (the networks were initialised with the same random seed). Weights in the Hebbian model are rather poorly tuned to the digit classes (e.g., the neuron resembling a “3,” “5,” and “8” in the second column of Figure 8A). On the other hand, DA-based learning leads to weights that more closely correspond to specific digits. This observation can be quantified by measuring the average responses of neurons to the different classes (first and third rows in Figure 8A). The measure shown indicates that Hebbian learning yields neurons exhibiting strong responses to multiple stimulus classes, i.e., with a broad tuning. Training with DA yields more sharply tuned weights as units respond almost exclusively to a single digit category.

On average over all neurons, DA generates a 17% increase in neurons' activations to their preferred classes, accompanied by a 84% reduction to non-preferred classes (Figure 7E). These modifications amount to neuron weights being more selective to specific digits, or having a sharper tuning. We quantify such neural selectivity as the difference between a neuron's mean response to stimuli of its preferred class and its mean response to stimuli of all other classes:

(13) $$\begin{array}{r} \zeta_{c}=\frac{{\overset{-}{s}}_{c}^{•}-{\overset{-}{s}}_{c}^{{}^{\circ}}}{{\overset{-}{s}}_{c}^{•}}, \end{array}$$

where ${\overset{-}{s}}_{c}^{•}$ and ${\overset{-}{s}}_{c}^{{}^{\circ}}$ are the average responses of neuron *c* to stimuli of its preferred and non-preferred classes, respectively. Here, ζ_*c*_ = [0, 1], where ζ_*c*_ = 0 is a neuron that responds equally strongly to all stimuli and ζ_*c*_ = 1 is a neuron that responds exclusively to one digit category. Selectivities of individual neurons are indicated on Figure 8A; selectivities averaged over all neurons of a network, ζ, are indicated on Figure 7E. We can also quantify a neural network's selectivity for a specific digit class *m* as the sum of the selectivity of the neurons whose preferred stimulus class is *m*, ζ_*m*_ (see Figure 8-B,C). Training with DA statistically significantly boosts neural selectivity (*p* < 0.001).

DA induces large improvements in classification accuracy (95.53±0.05% for DA compared to 83.5±0.7% for Hebbian learning, *p* < 0.0001), corresponding to a 72.7% reduction in the error rate. Performance for a class strongly correlates with neural selectivity for this class, for both the Hebbian and DA networks (*r* = 0.996 and *r* = 0.920, respectively, Figures 8B,C). These strong correlations suggest that enhanced neural selectivity explains the rise in correct responses following training with DA.

**Figure 9:**
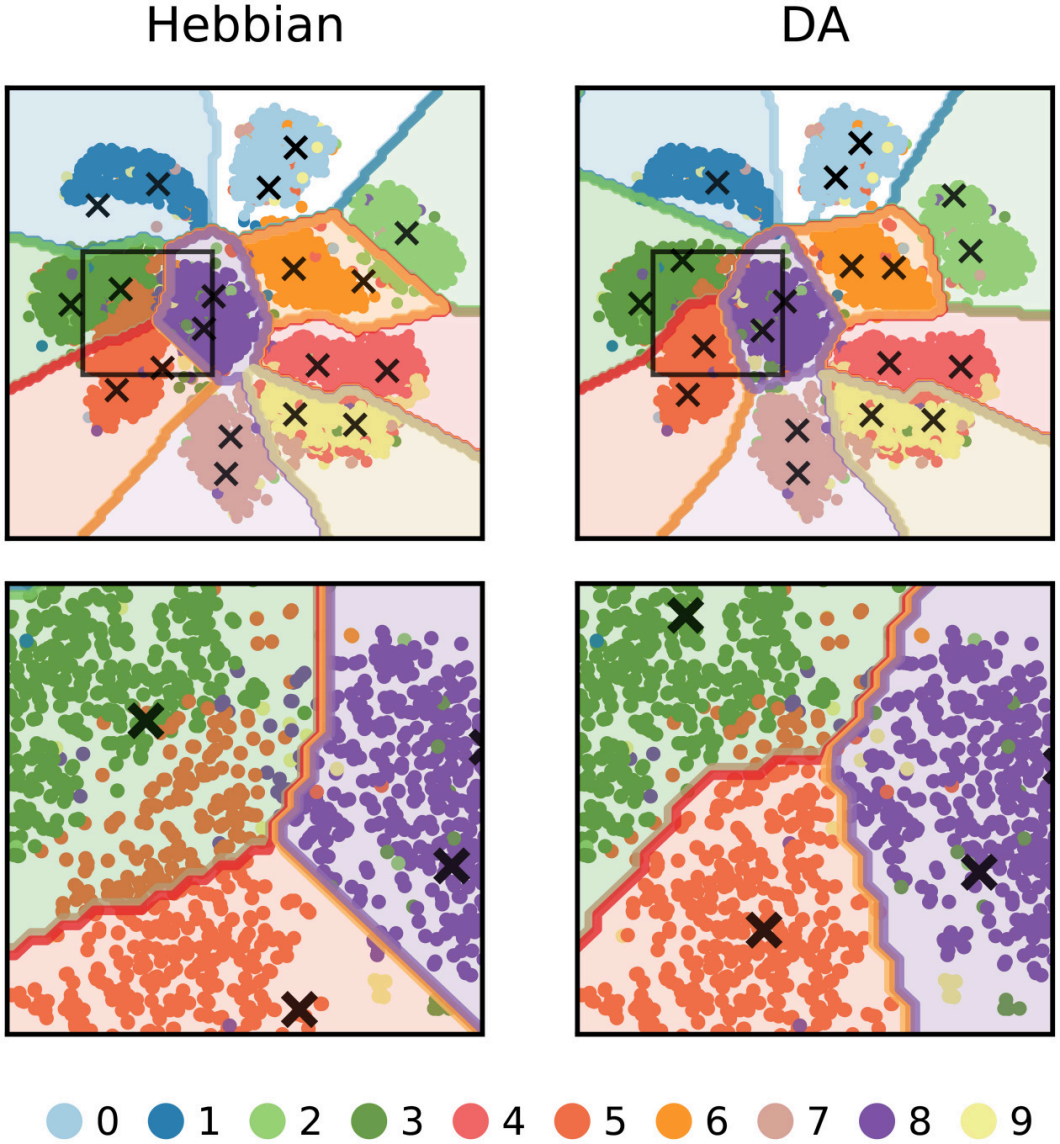
2-dimensional visualization of the outcome of DA learning. The dimensionality of input images are reduced from 784 to 2 features and a network is trained on these data. The input stimuli are depicted as colored dots, the weights of representation neurons as black crosses, and the classification boundaries as colored outlines. Hebbian learning performs density estimation: weights represent clusters of data points agnostic to the points' labels. For classes that are well separated from others, the network retrieves close to perfect boundaries (e.g., “1” or “0”). However, for close-by classes (e.g., “3”, “5”, and “8”, magnified in the bottom row), the boundaries poorly match the true labels. DA transmission adapts weights so that they better agree with the class boundaries of the task.

We can further visualize the outcome of DA learning by reducing the dimensionality of input images to 2 features (using t-SNE, Maaten and Hinton, 2008) and train the network on these data (Figure 9). In Hebbian learning, the neural network acts as a clustering algorithm and, as the learning mechanism is agnostic to the labels of the stimuli, the classification boundaries miss some aspects of the data classes. In particular, boundaries are poorly defined between close-by clusters such as “3,” “5,” and “8.” Following DA signalling, weights adjust to match the boundaries for the conditions for reward delivery of the task.

In the model for DA activation presented above, reward predictions are binary, reflecting solely whether a decision is explorative or not. An alternative approach is to use the classifier's posterior for the output class (i.e., its classification confidence) as an approximation to the expected value of the predicted reward. This posterior probability strongly correlates with the empirically-measured reward probability (*r* = 0.98), validating the approximation. However, we find that this approach does not improve the network's accuracy over binary reward predictions (Figure 5, “DA post”).

In order to assess the role of exploration in DA-based learning, we train a network without allowing explorative decision making. This greedy network achieves a classification score of 92.51±0.07% (Figure 5, “DA greedy”), compared with 95.53±0.05% with exploration. Exploration thus accounts for a further 18% relative drop in the error rate.

#### 3.2.4. Learning on non-uniformly distributed data

**Figure 10:**
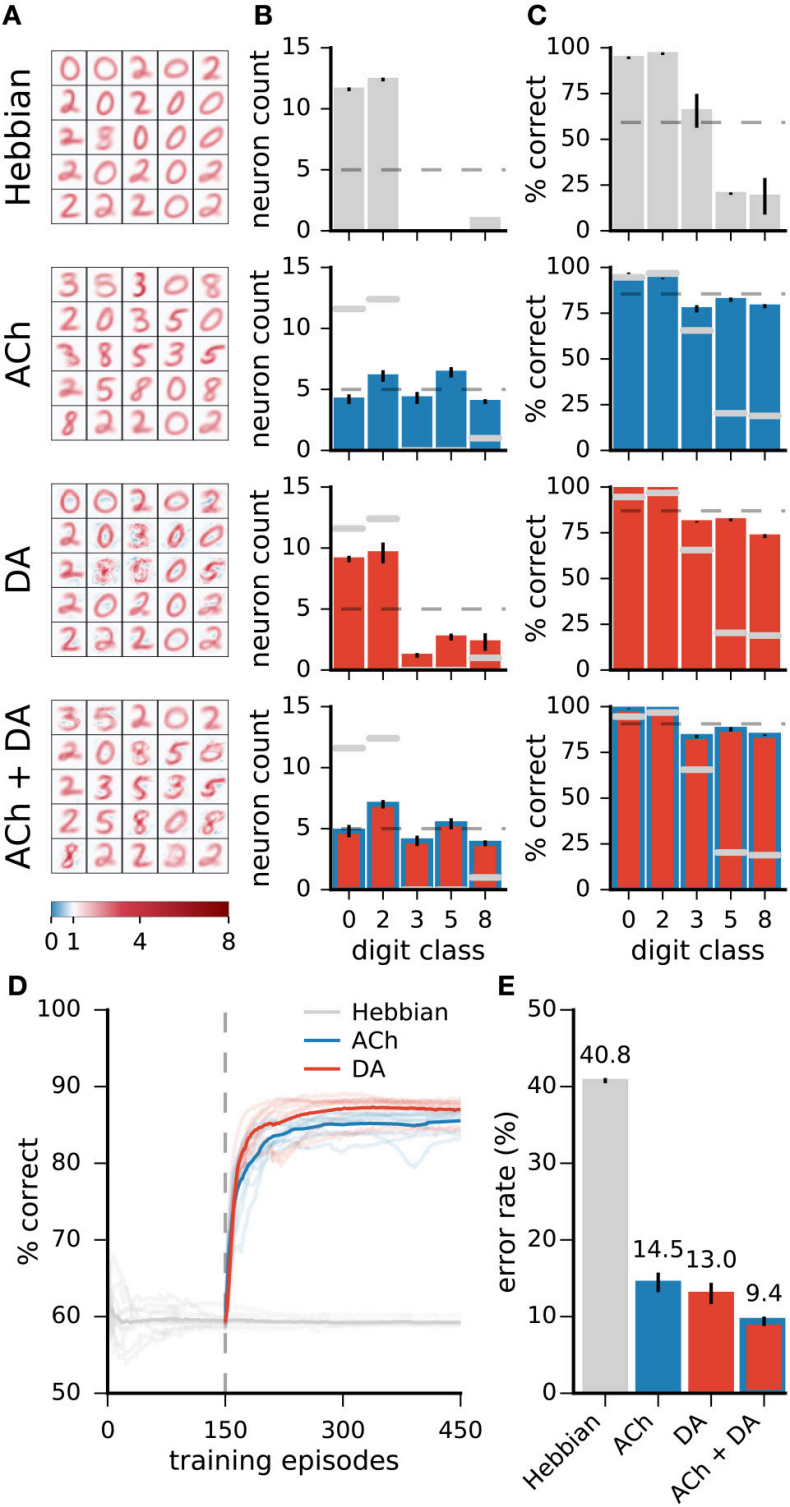
On non-uniformly distributed data, ACh and DA yield gains in accuracy of similar magnitudes. **(A)** Weights of the networks. **(B)** Distribution of the neurons' preferred digit classes. Dashed line is a uniform distribution. **(C)** Rates of correct classification on the test dataset. Dashed line is the mean over classes. **(D)** Progression of the test error for Hebbian, ACh, and DA. Lighter traces are individual runs, darker traces are the mean of 10 runs. **(E)** Error rates of the different methods. Data are the mean of 10 runs, error bars indicate the standard deviation, gray overlaid bars in **(B,C)** are data for the Hebbian network for comparison. On non-uniformly distributed data, ACh and DA bear effects of comparable magnitudes. The refinements in weights brought by the two modulators can combine to bring further decrease in error rates.

For the results on the MNIST dataset, ACh yields modest reductions in error rates relative to DA. This less important effect may be explained in part by the almost even distribution of training examples over the classes in the dataset. In more natural settings, some classes may contain many more examples than others while a high classification performance is equally important on all classes. For instance, a gatherer may see many more examples of “green leaves” than “berries” but still requires a low error rate for both classes. We test the impact of ACh in a modified version of MNIST in which a subset of the classes are over-represented. Here, the training dataset contains the classes “0,” “2,” “3,” “5,” and “8,” and there are 60 times more “0” and “2” (the “leaves”) than the other classes (the “berries”). To model equal importance of the classes, we take the test dataset to be uniformly distributed over the classes. For Hebbian learning, the network performs poorly on the under-represented classes as it dedicates only few neurons to these classes (Figure 10, top row). Neuromodulation significantly improves accuracy and, on these data, ACh yields gains comparable in size to those of DA. As with the standard MNIST dataset, ACh carries its effect by attributing more neurons to classes on which performance is low (those that are under-represented). DA only has minimal effects on the distribution of class preference; increases in performance derive from boosting neural selectivity.

In addition to training the network with ACh and DA separately, we combine the two neuromodulators by allowing first ACh release and then DA. This procedure leads to a redistribution of the class preferences (due to ACh) followed by an enhancement in neural selectivity (due to DA). The combined activations of ACh and DA result in a further decrease in error rates compared to either modulator alone, indicating that the effects of ACh and DA can successfully combine (Figure 10).

#### 3.2.5. Impact of code sparseness

**Figure 11:**
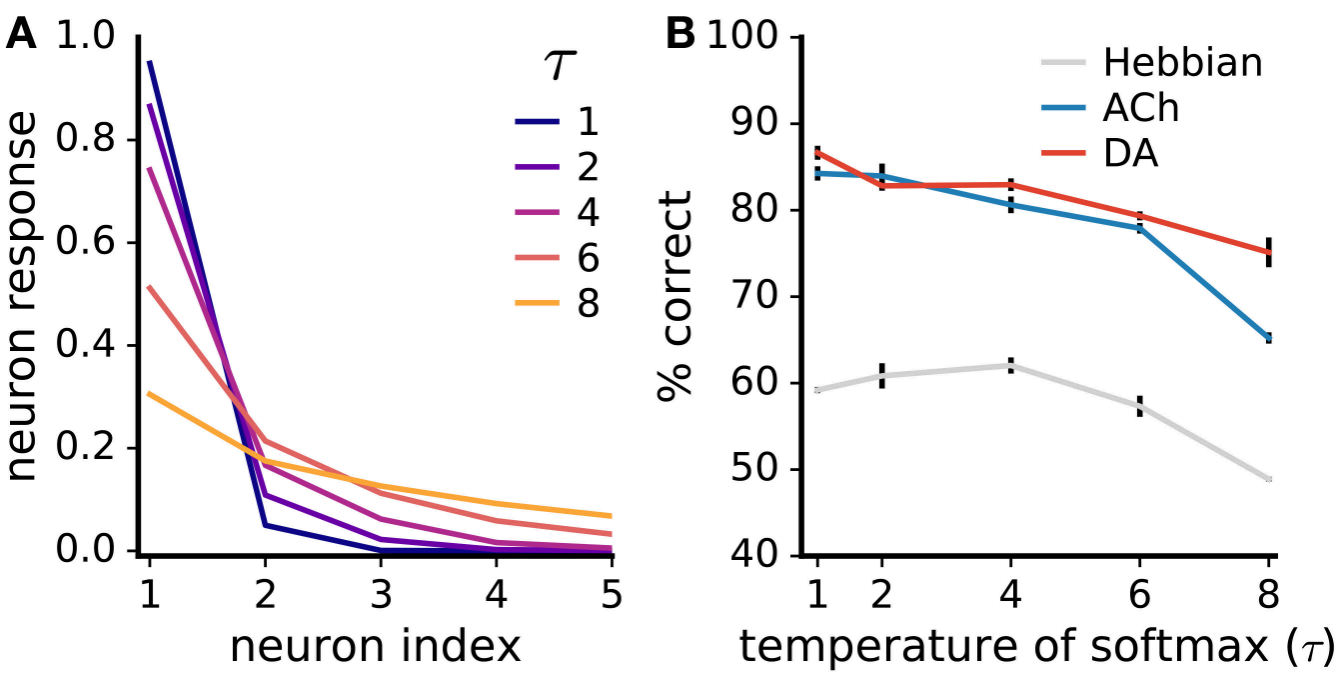
Neuromodulator-based learning improves performance also for low code sparseness. **(A)** Impact of the temperature parameter τ of the softmax function (Equation 14) on the sparseness of the neural code. The neuron indices are ordered from highest to lowest neural responses; the five most active neurons are shown. **(B)** Rate of correct classification for different τ values. Performance is on the non-uniform MNIST dataset. Data are mean of 3 runs, error bars are the standard deviation. Although performances drop with weaker competition, neuromodulators boost accuracy even for low code sparseness.

Lateral inhibition sparsifies the network's neural code so that inputs activate only one or a few neurons at a time (Figure 11A). Such a strong sparse code facilitates learning with neuromodulators as it avoids the credit-assignment problem. Additionally, the global neuromodulator signals are then essentially computed for a single neuron at a time. To examine the extent of the impact of the code's sparseness on learning, we introduce a temperature parameter τ to the softmax function determining the strength of the lateral competition:

(14) $$\begin{array}{r} s_{c}=\frac{\exp\left(I_{c}/\tau \right)}{\sum_{c\prime }\exp\left(I_{c\prime }/\tau \right)}. \end{array}$$

For τ → 0^+^, the softmax function gives rise to a winner-take-all competition with a single active neuron; for τ → ∞, neural responses are uniformly distributed. We train networks with different τ values on the non-uniform MNIST dataset (we use the non-uniform dataset to better discern the effects on ACh-based learning). We find that the networks' performance drops as code sparseness decreases (Figure 11B). However, the neuromodulators give rise to large and statistically significant improvements even for low code sparseness, indicating that strong competition is not required for effective neuromodulator-based learning.

#### 3.2.6. Impact of label availability

**Figure 12:**
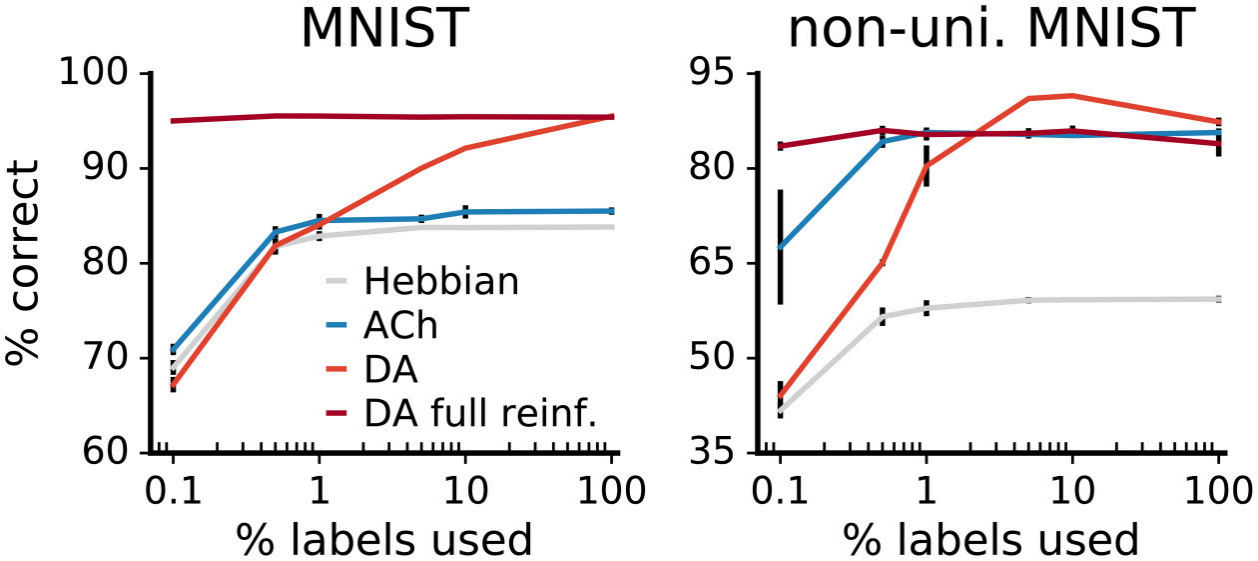
Comparison of label reliance for ACh and DA. Network performance as a function of the percentage of training labels used. For “DA full reinforcement” we use the indicated portion of labels to train the classifier but use all labels to provide the reward feedback. The size of the gains deriving from ACh are not statistically different from each others for any label fractions (*p* > 0.01). Data are the mean of 3 runs, error bars indicate the standard deviation.

We examine the impact of label availability on learning by training networks with a varying fraction of labels, from 100% down to 0.1%. The accuracies of the networks decrease with label scarcity, both for learning with Hebb's rule and with neuromodulators (Figure 12). For the Hebbian network, labels only affect the classification layer; the decay in performance therefore derives exclusively from lower classifier accuracy.

For the neuromodulators, while label scarcity affects them both, the consequences are more substantial for DA. In particular, when less than 1% of labels is used, the benefits of DA drop below those of ACh, this for both versions of the MNIST dataset. In error-based learning, labels are necessary to determine the correctness of an output. Reducing the ratio of labeled data consequently substantially hinders DA learning. On the other hand, the ACh signal yields gains in performance that are not statistically significantly different for all label fractions (*p* > 0.01). The constant improvements over declining label availability suggest that ACh learning relies effectively only minimally on labels, making ACh signaling beneficial even for scarcely labeled data.

DA-based learning does not require labels per se but only indications of whether outputs are right or wrong. We train an additional network using a fraction of the labels for the classifier but all labels for the reward feedback. The results show that performance remains high even for small label fractions, indicating that DA performs well in scenarios where true labels are in short supply but reinforcement feedback is available.

#### 3.2.7. Performance benchmark

In order to benchmark the functional performance of our algorithm, we compare it to MLPs trained with error back-propagation. We use the same architecture for our network and the MLPs (in this case, 784 input, 300 hidden, and 10 output neurons) and report the test error rate on the MNIST data. We train the MLPs using two state-of-the-art optimisation methods, the L-BFGS (Zhu et al., 1997) and Adam algorithms (Kingma and Ba, 2014) (see Appendix). In the original publication of benchmark results on the MNIST dataset, LeCun et al. (1998a) report a test error of 4.7% for an MLP of the architecture described above. Our biology-inspired algorithm yields a mean error rate of 2.88 ± 0.05%, outperforming this original result. The MLPs with the L-BFGS and Adam optimisers yield an error rate of 2.15 ± 0.04% and 1.88 ± 0.02%, respectively (Figure 5). In comparison, spiking neural networks intended for neuromorphic systems reach error rates of 5.0% (6,400 hidden spiking neurons, Diehl and Cook, 2015) and 4.4% (500 hidden spiking neurons, Neftci et al., 2015).

## 4. Discussion

### 4.1. Learning mechanisms

We study the effects of two modulatory signals on the representation and classification performance of a neural network. In our model, both signals act identically on synaptic plasticity but follow different release schedules, putatively those of ACh and DA. We find that these two signals give rise to distinct modifications in neural representations that both improve classification performance. Our model allows us to formulate hypotheses regarding the functional roles of ACh and DA in cortical representation learning. These roles can be explained as follows.

Consider the input ${\vec{y}}^{\left(n\right)}$ and the weights ${\vec{W}}_{c}$ as vectors in a high-dimensional space. The activation of a neuron *s*_*c*_ is computed as the dot product between an input and the weight vectors. Lateral inhibition introduces a soft winner-take-all competition resulting in a few neurons having strong responses and other neurons being silent. Hebbian learning then induces weight modifications $\vec{\Delta W_{c}}=\epsilon \cdot s_{c}\left(\vec{y}-{\vec{W}}_{c}\right)$ (Equation 5). We note that, for each weight, $\vec{\Delta W_{c}}$ points from the weight towards the current input. Both the variables *ACh* and *DA* modulate the magnitude of $\vec{\Delta W_{c}}$, $\left||\vec{\Delta W_{c}}|\right|$ (Equations 5a and 5b).

Hebbian learning in the network performs density estimation: the distribution of the weights is determined by the density of data points in the input space. Modulating the learning rate of the network is similar to modifying data point density in that presenting a training image twice is comparable to presenting this image once but with a twice larger learning rate. For ACh-based learning, input images that are more challenging will trigger greater ACh activation, or have a larger learning rate. A cluster of data points associated with greater ACh activation is thus similar to having more data points in this cluster, inducing more neurons to represent the cluster. Or in other words, data points with *ACh* > 1 will have $\left||\vec{\Delta W_{c}}|\right|$ of a greater magnitude, thereby exerting an increased “pull” on the weights.

**Figure 13:**
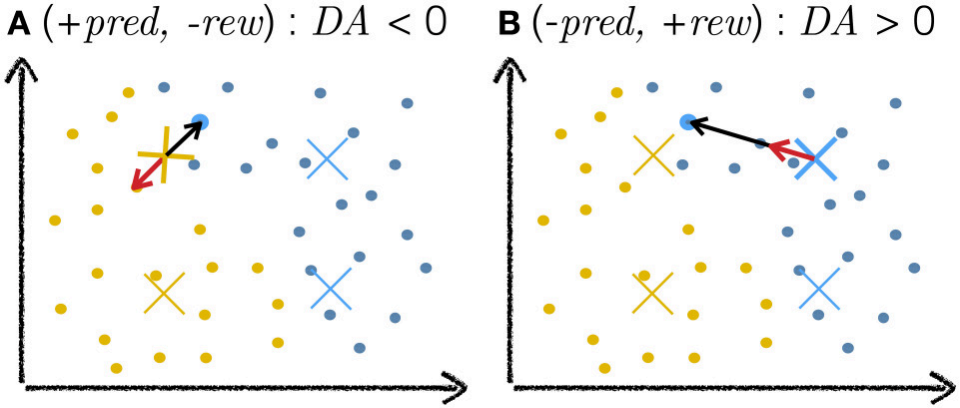
Cartoon explanation of DA-based learning. The plots depict a toy example of a two-dimensional input space with dots as training examples and crosses as neuron weights, the colors of which indicate classes. The highlighted blue training example is the current input to the network. The black arrow depicts the weight change vector $\vec{\Delta W}$. The red arrow depicts the same vector $\vec{\Delta W}$ after modification by the *DA* variable. As a scalar multiplier, *DA* only affects the magnitude (and sign) of $\vec{\Delta W}$ and leaves its direction unchanged. **(A)** The network makes an incorrect exploitative decision: the blue input activates the yellow cross. The network expected a reward but none is delivered. In this case, *DA* < 0, negating $\vec{\Delta W}$ and moving the weight away from the training example (red arrow). **(B)** The networks makes a correct explorative decision: the exploitative scenario would have activated the yellow weight near the current input but noise injection in the activation of neurons led to another (blue) neuron being more active. This decision is surprisingly correct and the network is rewarded. In this case, the value of *DA* is positive, moving the weight towards the current input (red arrow).

For DA-based learning, the variable *DA* takes a value δ_./._ specified by the current RPE scenario. According to the parameter search, for correct reward predictions (+*pred*, +*rew* or −*pred*, −*rew*), the optimal δ_+/+_ and δ_−/−_ are of approximatively zero. In both cases, $||\vec{\Delta W_{c}}||\approx 0$; all the network's weights remain unchanged. When the network takes an exploitative decision that turns out to be wrong (+*pred*, −*rew*), the optimal δ_+/−_ is inferior to zero. The vector $\vec{\Delta W_{c}}$ is negated so that it points away from the current input (Figure 13A). Active neurons will have their weights move away from the current input and are then less likely to win the softmax competition at future presentations of this input. When the network takes an explorative decision that is surprisingly correct (*-pred*, +*rew*), the optimal δ_−/+_ is positive. The weights of active neurons move towards the input (Figure 13B). The explorative decision (expected incorrect) turned out to be right; this decision should be taken again on future presentation of the same stimulus. DA-based learning can be understood as reinforcement learning at the level of sensory representations.

These learning mechanisms are related to several known machine learning algorithms. In the purely Hebbian case, the network is akin to a Kohonen map (Kohonen, 1982) in that learning proceeds iteratively through neural competition and weight adaptation (without however the cooperation aspect which confers the topological organization to Kohonen maps). The ACh learning mechanism is reminiscent of boosting methods, for instance AdaBoost (Freund et al., 1999), which attribute greater weights to misclassified training examples. The DA learning mechanism is closely related to algorithms such as REINFORCE (Williams, 1992) which make use of a reinforcement signal acting on the learning rate of a neural network's weight update rule. It is interesting to note that, despite this close correspondence, the decision to model DA as a modulation of the network's learning rate was made not to match those rules but rather to mirror biology. Indeed, our model of DA (and ACh) emulates the observation that stimuli coinciding with release of the neuromodulators are over-represented in animal sensory cortices (Figure 4). The close similarity between our model of DA and REINFORCE's learning rule can thus be taken as further support for the biological realism of the latter.

### 4.2. Acetylcholine

Activation of the cholinergic system in mammals appears to follow attentional efforts. Sarter et al. (2006) review evidence suggesting that deteriorating performances, as indicated by a rise in error rates and a decline in reward rates, trigger effortful cognitive control to prevent erroneous behavior. Attentional efforts are paralleled by a heightened activation of cholinergic neurons in the basal forebrain (Himmelheber et al., 2000; Passetti et al., 2000; Dalley et al., 2001; Arnold et al., 2002; McGaughy et al., 2002; Kozak et al., 2006) which in turn broadcast this signal to the cortical mantle (Hasselmo and Sarter, 2011). For instance, engaging in a demanding motor (Conner et al., 2010) or tactile (Butt et al., 1997) task enhances ACh release in the motor and somatosensory cortices, respectively.

There is broad evidence that ACh acts as a permissive plasticity agent at its projection sites (Buchanan et al., 2010; Giessel and Sabatini, 2010), for instance promoting alterations of neural representations in sensory cortices (Greuel et al., 1988; Bröcher et al., 1992; Kilgard and Merzenich, 1998a,b; Ji et al., 2001; Ma and Suga, 2005; Suga, 2012; Chun et al., 2013). The scientific literature contains several hypotheses regarding the functional role of the modifications elicited by ACh. Froemke et al. (2007) suggest that shifts in neural tunings toward a stimulus paired with ACh activation serves as a long-term enhancement of attention to this stimulus. Others postulate that this modification stores the behavioral relevance of the stimulus (Kilgard and Merzenich, 1998a; Weinberger, 2003) or generally improves signal processing (Gu, 2003; Froemke et al., 2013). Here, we show that a signal modulating synaptic plasticity as a function of task difficulty improves the quality of a neural representation with respect to a classification task. The gains in performance result from assigning more neurons to challenging stimulus classes. Our model suggests that ACh serves this role in mammalian cortices.

Experimental evidence offer support for this hypothesis. For instance, motor skill acquisition and the accompanying enlargement of relevant representations in the motor cortex require ACh activation (Conner et al., 2003, 2010). Conversely, discrimination abilities rise for a tone whose representation is expanded as a result of repeated pairing with ACh activation (Reed et al., 2011). More generally, ACh antagonists or lesion of the cholinergic system impairs perceptual (Butt and Hodge, 1995; Fletcher and Wilson, 2002; Wilson et al., 2004; Leach et al., 2013) and motor skill learning (Conner et al., 2003). These results indicate that the cholinergic system is crucial for forms of learning involving modifications in sensory maps, especially those affecting the relative extent of cortical representations, as suggested in this work.

Our model of ACh is in line with a previous simulation study by Weinberger and Bakin (1998). The authors make use of a modified version of Hebb's rule and simulate the action of ACh as an amplification in the post-synaptic activation of target neurons. An *in vivo* micro-stimulation study validates this model. For the Hebbian rule used in this work, the two models of ACh are mathematically equivalent; this previous work thus offers support to the simulation employed here.

### 4.3. Dopamine

Dopaminergic neurons of the midbrain encode various features of rewards (Satoh et al., 2003; Tobler et al., 2005) and, in particular, strongly respond to the difference between predicted and received rewards (Schultz et al., 1997; Schultz, 2010). Midbrain neurons project to the entire cortex (Haber and Knutson, 2010) and the reward signals they carry modulate neural activity in most cortical areas (Vickery et al., 2011) including primary sensory cortices (Pleger et al., 2009; Brosch et al., 2011; Arsenault et al., 2013).

DA affects plasticity at the sites where it is released, as measured both at the level of synapses (Otani et al., 1998; Centonze et al., 1999; Blond et al., 2002; Bissière et al., 2003; Li et al., 2003; Sun et al., 2005; Matsuda et al., 2006; Calabresi et al., 2007; Navakkode et al., 2007) and behaviorally (Brembs et al., 2002; Wise, 2004; Graybiel, 2005; Kudoh and Shibuki, 2006; Klein et al., 2007; Luft and Schwarz, 2009; Molina-Luna et al., 2009; Hosp et al., 2011; Schicknick et al., 2012; Ott et al., 2014). In sensory cortices, DA efflux, triggered either by electric stimulation of the midbrain or by reward delivery, elicits plastic changes in the responses of primary sensory neurons (Bao et al., 2001, 2003; Beitel et al., 2003; Frankó et al., 2010; Poort et al., 2015).

The role of the plastic modifications induced by DA are usually understood in terms of reinforcement learning, for instance to learn the appetitive value of stimuli (Brembs et al., 2002; Wise, 2004; Frankó et al., 2010) or to learn reward-directed behaviors (Watkins and Dayan, 1992; Dayan and Balleine, 2002; Wise, 2004; Schicknick et al., 2012; Ott et al., 2014). In sensory representations, the changes brought forth by DA were previously hypothesized to enhance the saliency of stimuli predictive of rewards (Bao et al., 2001) and to adapt cortical representations to task requirements (Brosch et al., 2011).

Here, we show that a signal modulating plasticity as a function of RPEs adapts synaptic weights to the reward contingencies of a task, thereby improving performance on the task. Specifically, in our model, the responses of neurons become matched to the boundaries in conditions for reward delivery. In the digit classification task, this results in neurons being better tuned to the distinct digit classes, in this way improving classification performance. We suggest that, in mammals, dopamine carries this role of adapting sensory representations to the reward contingencies of a task.

After training monkeys on a visual discrimination task, neural responses become matched to the stimulus features that discriminate between the reward conditions of the task (Sigala and Logothetis, 2002). This process is comparable to the effect of DA in our model. We thus postulate that DA orchestrates these changes and predict that lesioning the dopaminergic system would prevent this form of learning. Animal experiments show that interfering with DA signaling impairs sensory discrimination learning (Kudoh and Shibuki, 2006; Schicknick et al., 2012), supporting this prediction.

The optimal values of the δ_./._ constants we find through parameter exploration are in close qualitative agreement with the release properties of DA observed in primates (Schultz et al., 1997; Tobler et al., 2005) (Figure 6). Both in animals and in the present model, unpredicted rewards lead to a rise in dopaminergic activation while the absence of predicted rewards lead to a reduction in activation. Correctly predicted rewards leave dopaminergic activation essentially unchanged. The release values in the model were selected to maximize performance on a discrimination task. It is conceivable that the dopaminergic activation schedule in animals was similarly selected through evolutionary pressures to maximize perceptual abilities.

We tested the effect of explorative decision-making while training with DA and found that exploration yields an additional relative reduction of 18% in error rates. Studies show that human subjects actively engage in exploratory behavior when making decisions (Daw et al., 2006). Explorative decision-making is usually understood as a method to sample available choices with the prospect of discovering an option richer than the current optimum. Our model suggests that, in perceptual decision making, such explorative behavior may additionally serve the purpose of refining cortical sensory representations.

### 4.4. Comparing acetylcholine and dopamine

On the non-uniform dataset, ACh gives rise to improvements comparable in size to those of DA. This result highlights the relevance of ACh in scenarios where training examples are largely non-uniformly distributed over the classes, as is often the case in natural conditions. Furthermore, in contrast to DA, the ACh signal yields gains in accuracy of constant magnitude over decreasing label availability. This finding points to a particularly beneficial role for ACh when environmental feedback is scarce.

On the non-uniform dataset, the combined effects of the two neuromodulators are greater than either one separately. This result indicates that the weight modifications brought by ACh and DA are distinct and complimentary, and that they can successfully combine.

### 4.5. Functional performances and outlook

The learning mechanisms presented in this work yield error rates close to that of state-of-the-art optimisation methods used to train MLPs for comparable network architectures. Since evolutionary pressures must have favored well performing learning mechanisms in the brain, any candidate model of cortical learning must offer strong functional performances. Our model meets this criteria, making it a suitable model for learning in biological neural structures.

In line with recent studies of biologically-plausible learning (Keck et al., 2012; Nessler et al., 2013; Schmuker et al., 2014; Diehl and Cook, 2015; Neftci et al., 2015), we used correct classification as a measure of performance. This measure facilitates the study of the functional roles of neuromodulators and the comparison with previous work. Our neuromodulator-based learning method can be extended to tasks beyond classification, for instance by generaliz ing the softmax competition to k-winner-take-all (O'reilly, 2001) or soft-k-winner-take-all (Lücke, 2009) competition.

Even in the sole context of classification, however, our approach offers several interesting advantages. For instance, compared to the traditional approach of gradient descent on a classification error, neuromodulator-based learning requires a weaker supervision signal, making use of binary rewards instead of explicit labels. Additionally, our model learns even in the absence of environmental feedback through Hebbian learning. Finally, weight modifications are based on synaptically-local information and on two signals broadcasted identically to all neurons, which matches capabilities of biological neural networks.

On the functional side, learning with DA and ACh has been shown to decisively improve classification performance in our model system. Although it was not the main focus of this study, we note that very high classification performances even for relatively small networks (compare sizes in Diehl and Cook, 2015; Neftci et al., 2015) could be achieved using neuromodulation. The use of neuromodulation in spiking neural systems for neuromorphic chips (Diehl and Cook, 2015; Neftci et al., 2015) is therefore likely to result in performance gains. Similarly, neuromodulation is expected to further improve performance of novel hierarchical networks with Hebbian learning (Forster et al., 2016) which have a functional focus on learning from data with very few labels.

It is interesting to note that, since the initial publication of the MNIST dataset, advances in gradient-based learning resulted in continuous and substantial decreases in error rates. The biologically-inspired method presented in this work is at a relatively early stage and we may expect similar improvements from future research.

## Author contributions

RH carried out the simulations, analyzed the data, and designed the study with contributions of JL and KO. JL provided theoretical background and support for the neural network model. JL and KO helped revising the manuscript. All authors read and approved the final version of the manuscript.

### Conflict of interest statement

The authors declare that the research was conducted in the absence of any commercial or financial relationships that could be construed as a potential conflict of interest.

## A. Appendix

### A.1. Code and data

The model was written in the Python programming language and was run on a computer cluster. The code for the neural network is available at https://github.com/raphaelholca/hebbianRL. The original MNIST dataset is available at http://yann.lecun.com/exdb/mnist/. The dataset is randomly split into training and testing sets; the network's performance is reported on the testing images not seen during training. The network with 300 hidden units used for performance comparison with other work was trained with the full (unbalanced) dataset. For all other results, the datasets were balanced so that they contain the same number of examples for each digit class. This balancing has negligible effects on the results.

### A.2. Weight initialisation

We pre-compute the activation of input neurons $\vec{y}$ through Equation 1 for the whole training dataset. Learning proceeds through full iterations over the dataset during which $\vec{y}$ are presented in a random order to the network. Weights of representation neurons are initialised using the statistics of the input images. Specifically, we initialise the weights with the mean activation of input neurons taken over the whole dataset, with the addition of noise to break symmetry:

(A1) $$\begin{array}{r} W_{cd}=\mu \left(y_{d}\right)-\sigma^{2}\left(y_{d}\right)\cdot \eta_{\text{init }}, \end{array}$$

where μ(·) and σ^2^(·) are the mean and variance taken over all training images *N*, respectively, and η_init_ is noise drawn from a uniform distribution in the interval [0,0, 2.0). Activations propagate through the network as a succession of Equations 2, 4, 5. Values for all hyper-parameters were found through grid search (see Table A1).

**Table A1:** Hyper-parameters used in training the network. Values were determined through parameter exploration.

**Parameter**	**Description**	**Value**
α	Amplitude of the sigmoid function for ACh release	2.0
β	Slope of the sigmoid function for ACh release	20.0
δ_+/+_	DA activation for correctly predicted reward	0.01
δ_+/−_	DA activation for incorrectly predicted reward	−1.0
δ_−/+_	DA activation for unexpected reward	4.0
δ_−/−_	DA activation for correctly predicted absence of reward	−0.25
A	Normalization constant for feedforward inhibition	1.0 × 10^3^
ϵ	Learning rate	5.0 × 10^−3^
υ	Variance of the normal distribution of noise η	0.3

### A.3. Batch learning

To speed up computation, we train the network using mini-batches; weight updates are computed over batches of 50 training examples. Using mini-batches only negligibly affects representation learning and the network's performance.

In the case of DA-based learning, negative learning rates (for absent expected rewards, +*pred* −*rew*) could potentially result in negative weights. For biological realism and computational stability, we prevent this by excluding weight updates for a representation neuron *c* if any weight *W*_*cd*_ would become negative after the weight update. For the parameter set presented in Table A1 in Supplementary Methods, this rule only rarely prevents learning (~0.1% of all batch updates). However, when performing parameter exploration of the δ_./._ variables, some parameter sets lead to rapid decay to negative weight values, and this rule is then necessary to ensure computational stability.

### A.4. Comparison benchmarks

The MLP algorithm was obtained from the *Scikit-learn* module Pedregosa et al., 2011) (version 18.dev0, downloaded on 04/29/16). We used 3-fold cross-validation and grid search to determine the values of the hyper-parameters (see Table A2). The two optimisation methods used to train the MLP were the Adam and L-BFGS algorithms. Adam is a first-order stochastic optimisation method that uses individual adaptive learning rates for the different parameters. L-BFGS (Limited-memory Broyden-Fletcher-Goldfarb-Shanno) is a quasi-Newton method.

**Table A2:** Hyper-parameters for the benchmarking algorithms, as implemented in the *Scikit-learn* module.

**Parameter**	**Description**	**Value**
hidden_layer_sizes	Number of neurons in the hidden layer	300
activation	Activation function for the hidden layer	‘relu’
algorithm	Algorithm for weight optimisation	‘adam’ or ‘l-bfgs’
alpha	Regularisation term (L2 penalty)	1e-06
batch_size	Size of mini-batches for stochastic optimisation	200
learning_rate_init	Initial learning rate (Adam)	0.001
beta_1	Exp. decay for estimates of 1st moment (Adam)	0.8
beta_2	Exp. decay for estimates of 2nd moment (Adam)	0.9
epsilon	Value for numerical stability (Adam)	1e-08
